# Reproducible 3D bioprinting of *Streptococcus mutans* to create model oral biofilms

**DOI:** 10.1128/spectrum.00935-25

**Published:** 2025-10-10

**Authors:** Guilherme Roncari Rocha, Danielle S. W. Benoit, Anne S. Meyer

**Affiliations:** 1 Knight Campus for Accelerating Scientific Impact Bioengineering Department, University of Oregon, Eugene, Oregon, USA; 2 Department of Biomedical Engineering, University of Rochester 3265, Rochester, New York, USA; 3 Materials Science Program, University of Rochester, Rochester, New York, USA; 4 Department of Biology, University of Rochester 171423, Rochester, New York, USA; Reichman University, Herzeliya, Israel

**Keywords:** Dental caries, biofilms, *Streptococcus mutans*, bio-ink, hydrogel, 3D-bioprinting

## Abstract

Novel approaches are needed to study relationships between oral biofilm strains, enable three-dimensional oral biofilm deposition, and hasten the rigor and pace of basic and translational biofilm studies. Previously, 3D-bioprinters were leveraged to deposit spatially patterned biofilms onto sugar-rich agar surfaces to study how the underlying spatial organization of various microbes impacts biofilm persistence and virulence. Herein, we have developed a new method to adapt this process from limited, soft agar surfaces to biomimetic solid substrates submerged in aqueous solutions for studying oral biofilms *in vitro. Streptococcus mutans* UA159 was used to compare standard *in vitro* biofilm development with our new 3D-printed bio-ink hydrogels on hydroxyapatite disks, which mimic tooth surfaces. Biofilms formed using the bio-ink methodology showed minimal quantitative differences in virulence factors, including environmental pH, biomass, and cell density, compared to biofilms formed using the standard *in vitro* methodology. The bio-ink technique resulted in higher exopolysaccharide deposition, a key virulence factor for biofilm cohesion and protection, as well as more homogeneous spatial distribution of bacterial microcolonies. Our newly developed technique produces 3D-printable model biofilms that match the virulence benchmarks of the standard method, opening possibilities to print biofilms onto any substrate and a new way to study multidimensional biofilm dynamics.

**IMPORTANCE:**

Dental caries is the most common oral disease caused by biofilms in humans with cost limitations. Changes in the human diet have increased the exposure to sugar-rich processed food, increasing the incidence and severity of dental caries and creating greater rationale for understanding biofilm deposition, microbial interactions, and maintenance of quiescence of the oral microbiota. Recent 3D-printing techniques have been leveraged to develop the first model biofilms, providing spatial control over microbe deposition and enabling unprecedented investigation of the impact of cell-cell interactions and spatial organizationupon biofilm persistence, sensitivity to drugs, and virulence. Here, we have developed new methods to extend bioprinting to oral biofilms using cariogenic *Streptococcus mutans*. Our technique is an attempt to establish an alternative method for oral biofilm formation *in vitro* that uses 3D-printing tools, preserving the virulence of standard *in vitro* biofilms while amplifying the availability and versatility of methods for understanding the microbiome.

## INTRODUCTION

** **Biofilms are a highly dynamic community of microorganisms embedded in a self-generated extracellular matrix (1–3). The predominance of certain microorganisms in the biofilm leads to changes in specific internal characteristics of the biofilm community, including extracellular matrix density, permeability, and viscoelasticity. These internal changes can impact the virulence of biofilms (4–6). One of the most prevalent diseases caused by biofilms in humans is dental caries, affecting 3.5 billion people, resulting in an annualworldwide cost of approximately $387 billion (7). The accumulation of microorganisms on the mineral surfaces of teeth, in combination with exposure to high sugar concentrations, increases the production of acid by caries-related oral biofilms. Development of dysbiotic oral biofilm is particularly prevalent when combined with other internal factors, including poor dental hygiene caused by infrequent or poor techniques in tooth brushing, minimal use of fluoride, etc., and external factors, such as education, socioeconomic factors, systemic diseases, and lack of or poor insurance coverage (2, 4, 8). These multifactorial circumstances can cause changes in environmental pH and oxygen tension, causing an imbalance between the population of strains dwelling in these communities. The sum of these internal and external factors can increase the deposition of the extracellular matrix, protecting and giving more cohesion and viscoelasticity to biofilms. Consequently, the biofilm matrix will reduce saliva buffering, increasing local acid accumulation and promoting teeth demineralization, also known as dental caries (9–12).

 *Streptococcus mutans* is a bacterial species prevalent in mature, dysbiotic oral biofilms (5) and is, therefore, the most-studied oral biofilm species. *S. mutans* predominates in caries because it has acid tolerance response mechanisms that allow it to survive in low pH environments by controlling cytoplasm acidification (5, 12–15). Greater acid production upon exposure to a sugar-rich diet also favors its predominance within caries-related biofilms (9, 15), resulting in the creation of larger deposits of extracellular matrix and disrupting the demineralization-remineralization process (8, 13, 14). Early colonizers, including *Streptococcus gordonii*, *Streptococcus sanguinis*, and others, also play an important role on the biofilm deposition process. These species serve as the first layer of microorganisms that use adhesins to interact with the salivary pellicle on the hydroxyapatite surface, thereby creating new binding sites for late colonizers like *S. mutans (*16, 17). However, environmental changes like high sugar exposure, oxygen tension, or changes in the pH may alter population predominance, microbial metabolism, and spatial distribution of microorganisms in the biofilm (18, 19). *S. mutans* uses F-ATPase and arginine deiminase systems to overcome the acid challenges and oxidative stresses faced during biofilm maturation and can become the predominant population in mature caries-related model biofilms *in vitro (*15, 20, 21). Most of the *S. mutans* population lives in the core of the biofilm protected by the strains more sensitive to environmental variation that live on the biofilm boundaries and near the extracellular matrix (20, 22, 23). In summary, caries-related dental biofilms are a highly dynamic multi-species community of microorganisms protected by a viscoelastic, nonpermeable matrix in which topical treatments such as saliva buffering or drugs cannot reach the bacteria on the core of mature biofilms due to low permeability, attacking only the more susceptible strains in the periphery (17, 20, 23).

The extracellular matrix of oral biofilms plays crucial roles in microbial protection, facilitating microbial interaction or co-aggregation, and serving as a source of nutrients (11, 17, 22, 24, 25). It largely comprises polysaccharides (polymeric chains made up of glucans), proteins, lipids, nucleic acids, lipopolysaccharides, extracellular DNA, and lipoteichoic acid (22, 25, 26). The internal bacterial population and biochemical environment of a biofilm can influence the production and deposition of each of these components (27, 28). In the presence of sugar, glycosyltransferase enzymes produced by *S. mutans* result in the production of glucans with α1-3-linkages, which are highly viscoelastic, less permeable, and alkali soluble; glucans with α1-6-linkages are comparatively less viscoelastic, more permeable, and water-soluble compared with α1-3-linkages; and fructans (4, 29–31). Therefore, a mature cariogenic biofilm containing more *S. mutans* is a challenge to remove either via mechanical brushing due to its high viscoelasticity or via topical treatments due to its low permeability (25, 32–34).

**Fig 1:**
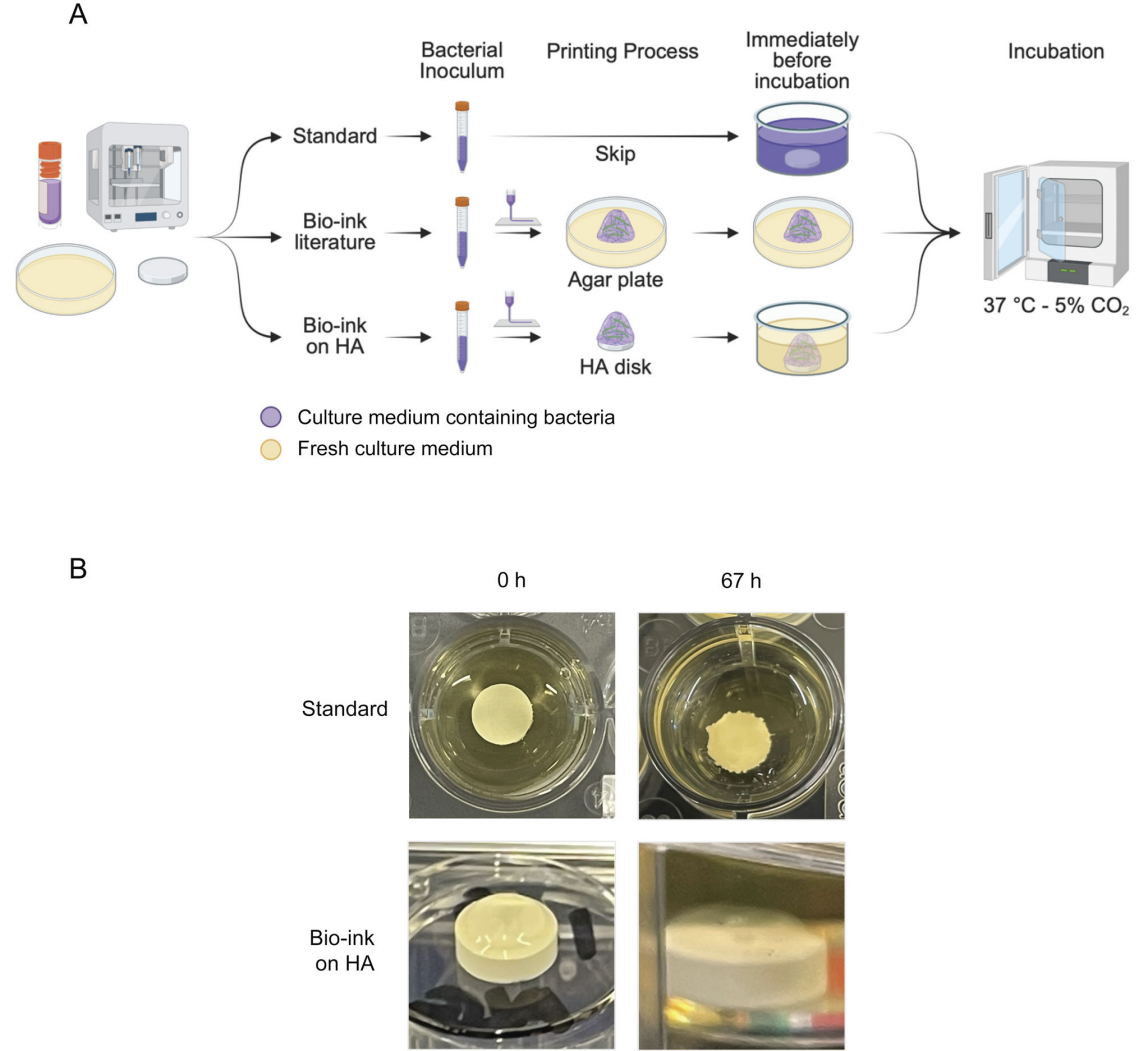
Generation of 3D-printed oral biofilms on hydroxyapatite surfaces. (**A**) Scheme showing the methodological differences between the preparation of standard biofilms (upper), a previously developed bio-ink methodology (middle, not analyzed here) (42, 43, 47, 48), and the novel bio-ink methodology tested here (lower) (created in https://BioRender.com). Standard model biofilms are formed by submerging a hydroxyapatite disk within the microbial culture (purple) to allow for bacterial adhesion and biofilm deposition on the substrate over time. The previous bio-ink methodology facilitates deposition of bacteria-containing bio-ink (purple) onto agar plates. The new bio-ink methodology facilitates deposition of bio-ink biofilms onto hydroxyapatite discs, which are then submerged within fresh culture media supplemented with 0.1 M calcium chloride. (**B**) Photographs of standard (upper) and bio-ink (lower) biofilms, showing the disks at 0 and 67 h. The hydrogel structure of the bio-ink biofilms was maintained throughout the incubation period.

 Screening new natural or synthetic products that can mitigate biofilm formation without creating bacterial resistance and that can penetrate or disrupt the challenges of the extracellular matrix is critical to develop more effective approaches to combat caries-related oral biofilms (23, 35). Current standard methods have been designed to develop biofilms *in vitro* to study biofilm biology, characterize bacterial interactions, and screen potential drugs. For these methods, the bacterial inoculum is prepared, a substrate such as natural tooth, synthetic hydroxyapatite disks, or other material of interest is exposed to the inoculum, and the samples are incubated under static or shaking conditions over time to allow for biofilm formation (Fig. 1) (23, 36). 3D-printing of microbes embedded within bio-ink is a new methodology developed in the last decade to automate the process of biofilm formation *in vitro (*37). In straightforward alginate-based bioprinting, the bio-ink contains the microorganisms, sugar, and hydrogel based on alginate. The bio-ink gelation process begins upon exposure to divalent cations, such as calcium (38–40). Alginate forms ionic crosslinks in a rapid concentration-dependent process (38, 41). This rapid hydrogel formation is an advantage for 3D-printed and controlled deposition of biofilm-forming strains to understand biological mechanisms of biofilm formation and develop targets for biofilm management (41–43). The development of 3D-printing technologies for microbes has enabled control over deposited single and multilayer strains (42–44) and can allow microbial viability and growth as well as development of biofilm phenotypes (42, 44–46).

Despite the opportunities inherent in 3D bioprinting of biofilm-forming microbes, 3D-bioprinting has not been applied to study oral biofilms. Additionally, the well-established technique of bioprinting of biofilms has been limited to agar surfaces as a primary source of sugar for biofilm growth (43, 44). However, to reproduce an oral environment, several challenges must be overcome to adapt the 3D-bioprinting technique for different substrates that better emulate oral cavities. The challenges faced include the following: (i) to deposit the biofilm onto hydroxyapatite (HA), a non-sugar mineralized surface that mimics tooth surfaces; (ii) to create a suitable environment for microorganisms in which they can be fed properly with sucrose after the sugar-rich agar surface has been replaced with a hydroxyapatite mineral surface; (iii) to develop a hydrogel that can maintain its structure upon extended exposure to aqueous media, while also allowing for the microorganisms to organize the community, creating deposits of extracellular matrix inside the hydrogel; (iv) the biofilm community must display virulence characteristics that are comparable with the well-established standard biofilms used in previous *in vitro* studies (Fig. 1A) (10, 13, 23, 36, 49, 50).

Therefore, in this work, we investigated whether the 3D microbial bioprinting technology could be used to develop model cariogenic biofilms. The new methodology leverages 3D-bioprinting of the microorganism *Streptococcus mutans* UA159, a model for *in vitro* single-species oral biofilm cariology studies, onto hydroxyapatite and compares the resultant 3D-printed biofilms to the standard method for creating oral biofilm deposits. Virulence factors that characterize a cariogenic biofilm, including dry weight, bacterial population, environmental pH, exopolysaccharide matrix content, and bacterial 3D distribution over time, were measured to compare the two approaches to developing cariogenic biofilms. This innovation may open a variety of research opportunities to study biofilms on hydroxyapatite using 3D-bioprinting.

## RESULTS AND DISCUSSION

### Development of a novel technique for 3D-printing oral biofilms onto hydroxyapatite surfaces

Microbial bioprinting has opened new avenues to studying different mechanisms of biofilm development and maturation *in vitro (*42, 43, 45, 47) (Fig. 1A). Expanding the possible 3D-printing substrates beyond the limits of agar surfaces may enable emulation of environmentally or biomedically relevant biofilms that have unique spatial organizations underpinning their function, such as model intestinal biofilms that include three-dimensional interaction between the constituent microorganisms (51). The primary goal of this project is to create a 3D-bioprinted method that can produce model oral biofilms that display similar biological and virulence phenotypes to oral biofilms created using the standard method currently used (Fig. 1A).

To develop a technique for 3D-bioprinting oral biofilms onto tooth-mimicking hydroxyapatite surfaces submerged within aqueous media, several modifications were implemented to the previously developed alginate-based biofilm printing techniques. First, the 3D-printed bacteria were cultured within a growth media containing the alginate used to form the bio-ink’s supportive hydrogel polymeric network, rather than supplementing the alginate into the culture medium following bacterial growth, thereby preventing dilution of the bio-ink culture medium during bio-ink preparation (42–44, 47). Second, the biofilm culture medium used for submerging the 3D-printed bio-ink samples was enriched with 0.1 M of calcium chloride crosslinking agent, allowing the hydrogel structure to remain submerged within the liquid culture medium for extended periods without depolymerizing or drying during culture, although natural biofilm development does not require this supplementation to occur. Third, to prepare the hydroxyapatite disks for printing, they were incubated briefly in calcium chloride solution to impregnate them with the crosslinking agent, enabling solidification of the alginate bio-ink upon deposition analogous to previously described methods for 3D-bioprinting onto calcium chloride-containing agar plates (42). Printing was followed by a short incubation (5 min) to allow hydrogel gelation, after which the hydroxyapatite disks with the bio-ink on top were carefully immersed within the liquid culture medium to allow for biofilm development (Fig. 1A).

*Streptococcus mutans* UA159 single-species biofilms were prepared in duplicate for a minimum of three different experiments and analyzed after 43 h and 67 h by depolymerizing the alginate hydrogel with sodium citrate (42). In an attempt to standardize the 3D-bioprinted bacterial inoculum, bio-ink was prepared containing *Streptococcus mutans* at 10^7^ CFU/mL (Fig. 1A), determined from an experimentally derived standard curve, and extruded onto hydroxyapatite disks. The same initial bacterial pre-inoculum culture that was prepared to create the bio-ink was also used to create all controls that used standard biofilm deposition, in which hydroxyapatite disks were directly exposed to liquid microbial culture at equivalent microbial concentrations (Fig. 1A) (13, 23). Two additional control conditions were utilized, standard biofilms grown with calcium chloride supplementation in the culture medium and standard biofilms post-treated with sodium citrate, to analyze any impact of incubation with calcium chloride crosslinking reagent or from the depolymerizing post-treatment with sodium citrate. The experimental images at 0 and 67 h demonstrate that the bio-ink biofilm was visible on the HA disks and that the hydrogel structure was maintained over the 67 h incubation time (Fig. 1B), likely due to the calcium supplementation.

### 3D-printed oral biofilms support bacterial growth

**Fig 2:**
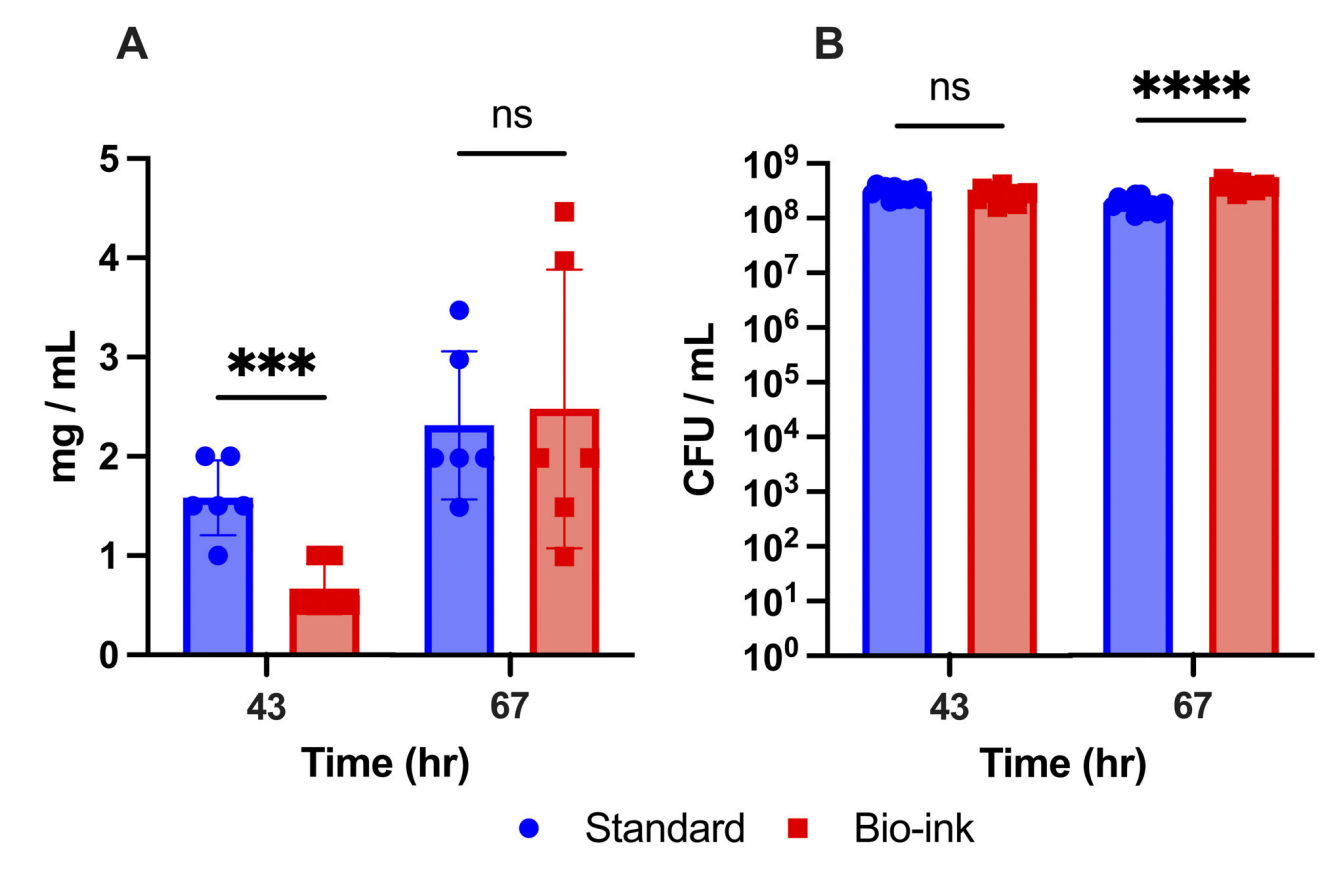
3D-printed oral biofilms support bacterial growth over time. (**A**) Insoluble dry mass of standard and bio-ink biofilms. (**B**) Population of viable *S. mutans* in standard and bio-ink *S. mutans* biofilms. Data are shown as mean ± standard deviation from three independent experiments in duplicate using ANOVA with Tukey’s correction for multiple comparisons. **** indicates *P* ≤ 0.0001, *** indicates *P* ≤ 0.001, and ns indicates no statistical difference. CFU/mL, colony-forming units per milliliter.

To determine the ability of 3D-printed *S. mutans* bacteria to survive and proliferate within the hydrogel, the insoluble dry mass was measured at 43 and 67 h of development (Fig. 2A). This assay indicates the amount of biomass within the biofilms, including live or dead cells, exopolysaccharide matrix, proteins, etc (13, 29). At 43 h, significantly less dry mass was observed for the bio-ink biofilm samples (≤66.6%, *P* ≤ 0.001) versus the standard biofilm (Fig. 2A). The dry mass of the standard biofilms that had been depolymerized with sodium citrate at 43 h was also significantly lower than that of the standard biofilms while being statistically indistinguishable from that of the bio-ink biofilms (Fig. 2A and Fig. S1A). These results indicate that the lower biomass in the bio-ink samples may have been attributable to the sodium citrate post-treatment employed to depolymerize the alginate during biofilm collection and processing, which is consistent with the findings of previous literature showing that sodium citrate treatment of 3D-printed biofilms at early time points results in significantly decreased bacterial viability (43). Calcium treatment of the standard biofilms was not observed to create a significant difference in biomass compared to the standard biofilms (Fig. 2A and Fig. S1A, standard versus control calcium), demonstrating that the calcium supplementation in the bio-ink samples likely did not interfere with the biofilm formation.

At 67 h, increased dry weights were measured for all samples, and no statistical differences were identified in dry mass between bio-ink and standard biofilms (*P* ≥ 0.05) (Fig. 2A) or versus any other control samples (Fig. 2A; Fig. S1A). The relatively larger standard deviation seen for the bio-ink samples compared to the standard samples (Fig. 2A) may indicate higher variability in biofilm development in the bio-ink condition. The mature biofilms at 67 h may have been less affected by the sodium citrate treatment in comparison to the younger samples at 43 h due to the increased amounts of extracellular matrix, which is associated with resistance to sodium citrate in 3D-printed biofilms (43, 47) and which can protect, stabilize, and confer viscoelasticity to biofilm structures (1, 12, 52). This result indicates that longer incubation periods for the 3D-bioprinted biofilms resulted in stronger biofilm community development that was directly comparable to that of the standard biofilms, with no measurable impact on the dry weight measurements of the mature biofilms by the post-processing treatment with sodium citrate.

To measure the size of the bacterial population in the biofilm samples over time, we determined the number of colony forming units (CFUs) in the biofilms at both 43 and 67 hours (Fig. 2B). High CFU levels (≥10^8^ CFU/mL) were seen for standard and bio-ink biofilms at both timepoints, and no statistical differences were found between bio-ink biofilms and any of the control groups tested (Fig. 2B; Fig. S1B). These results indicate that the bio-ink biofilms were able to support *S. mutans* survival and growth at rates comparable to that of the standard model. Additionally, the similarity in CFU levels across conditions indicates that the differences seen in the biomass of standard versus bio-ink biofilms at 43 h did not derive from differences in the number of living bacteria within the communities at that time point. The variance in biomass could instead be related to the sodium citrate post-processing treatment or to differences in the production of extracellular matrix components.

### 3D-printed oral biofilms deposit increased exopolysaccharide matrix

The 3D-printed *S. mutans* biofilms were quantitatively analyzed for their ability to deposit exopolysaccharides. One of the most important virulence factors is the deposition of the extracellular matrix, the structure of which provides structural cohesion and stability, protection from environmental insults, and a nutritional source in conditions of sugar shortage (9, 11, 25). The most abundant component of the extracellular matrix is exopolysaccharides (52, 53), made of polymerized glucans and fructans (52). Polysaccharide abundance can be determined quantitatively by extracting either the water-soluble polysaccharides (WSP; predominantly α1-6-glucan linkages) or the alkali-soluble polysaccharides (ASP; predominantly α1-3-glucan linkages) and measuring their absolute abundance using a phenol-sulfuric acid assay (54).

**Fig 3:**
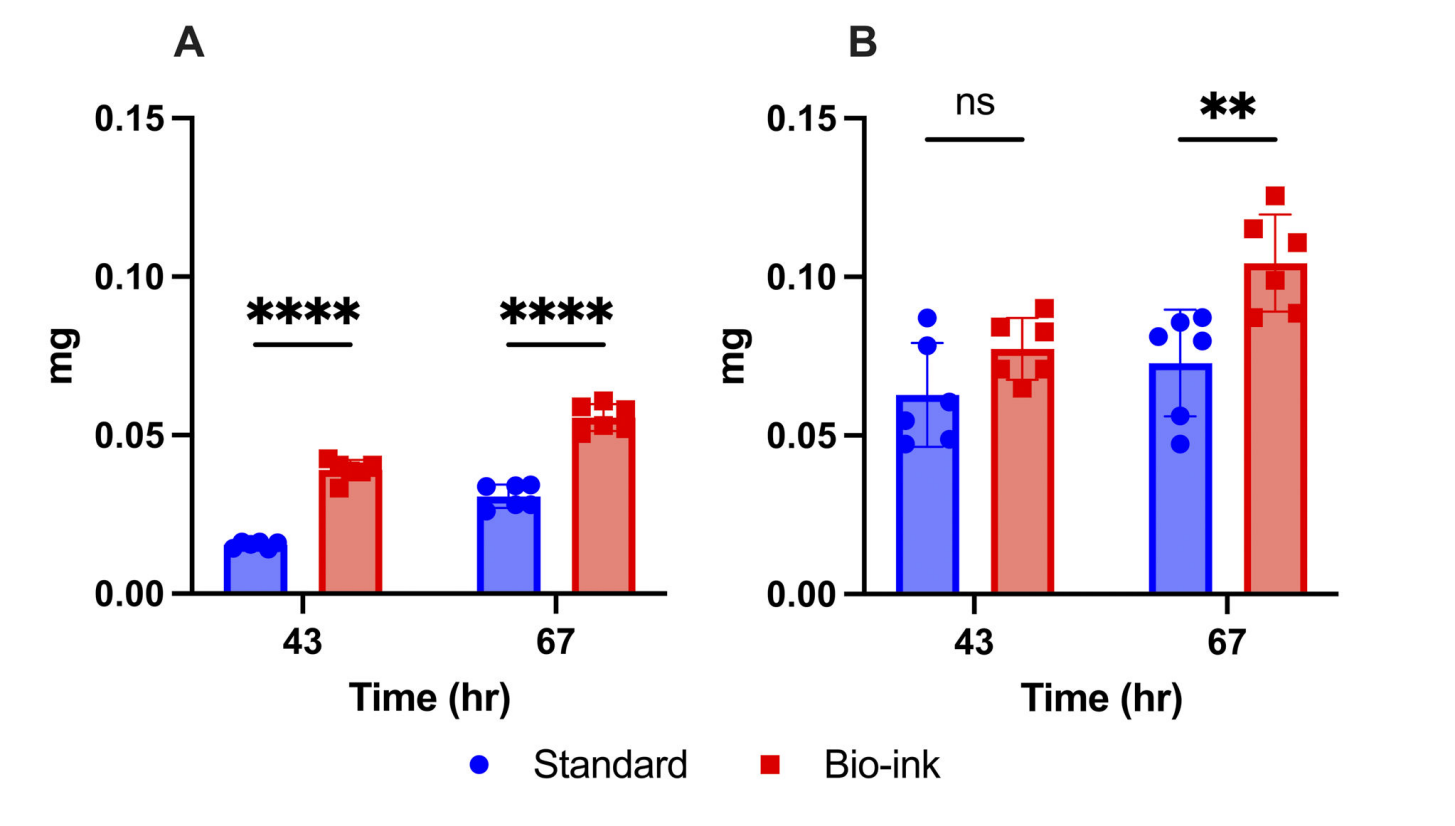
3D-printed oral biofilms deposited a greater amount of exopolysaccharides. (**A**) Water-soluble exopolysaccharides and (**B**) alkali-soluble exopolysaccharides of standard and bio-ink *S. mutans* biofilms. Data are shown as mean ± standard deviation from three independent experiments in duplicate using ANOVA with Tukey’s correction for multiple comparisons. **** indicates *P* ≤ 0.0001, ** indicates *P* ≤ 0.01, and ns indicates no statistical difference.

Quantification of the water-soluble polysaccharide content of the different samples indicated that the bio-ink biofilms deposited significantly higher amounts of WSP compared to the standard biofilm samples at both time points (Fig. 3A). The bio-ink biofilms contained approximately twice as much WSP than the standard biofilm samples tested (Fig. 3A; Fig. S2A). This increased amount of WSP is predicted to increase the biofilm cohesion, as well as the difficulty for topical treatments to penetrate and target the microbial community in these 3D-printed biofilms. Two additional control conditions were utilized: standard biofilms grown with calcium chloride supplementation in the culture medium and standard biofilms post-treated with sodium citrate, to analyze any impact of incubation with the calcium chloride crosslinking reagent or from the depolymerizing post-treatment with sodium citrate. The WSP deposition for the standard biofilms was not significantly different from either of the control groups (Fig. S2A), indicating that calcium supplementation and sodium citrate post-processing did not contribute to the increased WSP deposition in the bio-ink biofilms.

Quantification of the alkali-soluble polysaccharide content of the biofilm samples showed that the bio-ink biofilms contained a similar amount of deposited ASP at the 43 h time point (*P* = 0.20) and a statistically higher amount of deposited ASP at the 67 h time point (*P* = 0.003) compared to standard biofilms (Fig. 3B). Analysis of the control samples indicated that calcium ion supplementation in the culture medium did not interfere with exopolysaccharide deposition (Fig. S2B). The sodium citrate post-treatment resulted in decreased ASP amount at 43 h, but not at 67 h (Fig. S2B), showing that the difference observed in the bio-ink biofilm dry weight at 43 h can be at least partially related to exopolysaccharide components. The significant reduction of ASP amount seen for sodium citrate-treated control samples at 43 h may indicate that the bio-ink samples produce more ASP at this early time point than was able to be measured due to sample loss during post-processing. The total amount of ASP deposited by each type of biofilm was nearly two times higher than the WSP content. As an additional control, the bio-ink alginate hydrogel by itself, without added microorganisms, was also tested in the WSP and ASP assays, which revealed no detectable levels of WSP and ASP, indicating that the alginate-based bio-ink by itself did not impact these tests.

ASP matrix polymers have higher viscoelastic and impermeability characteristics compared to WSP (26), indicating that the bio-ink *S. mutans* biofilms expressed pathogenic characteristics (15) (Fig. 3). The overall higher exopolysaccharide content of the bio-ink biofilms implies that they will pose a greater challenge to the permeability and effectiveness of topical treatments relative to the standard biofilms. These bio-ink biofilms are also expected to display an increased biofilm cohesion that facilitates microbial co-aggregation, as well as high deposition of nutrient sources for the embedded microbes. If this method is used for testing the drug efficiency in future studies, the adaptive stress responses elicited by the application of topical treatments may result in increased polysaccharide deposition, possibly decreasing the effectiveness of the topical treatments due to greater exopolysaccharide content that can directly impact diffusion (11, 32).

### 3D-printed oral biofilms acidify their environment

**Fig 4:**
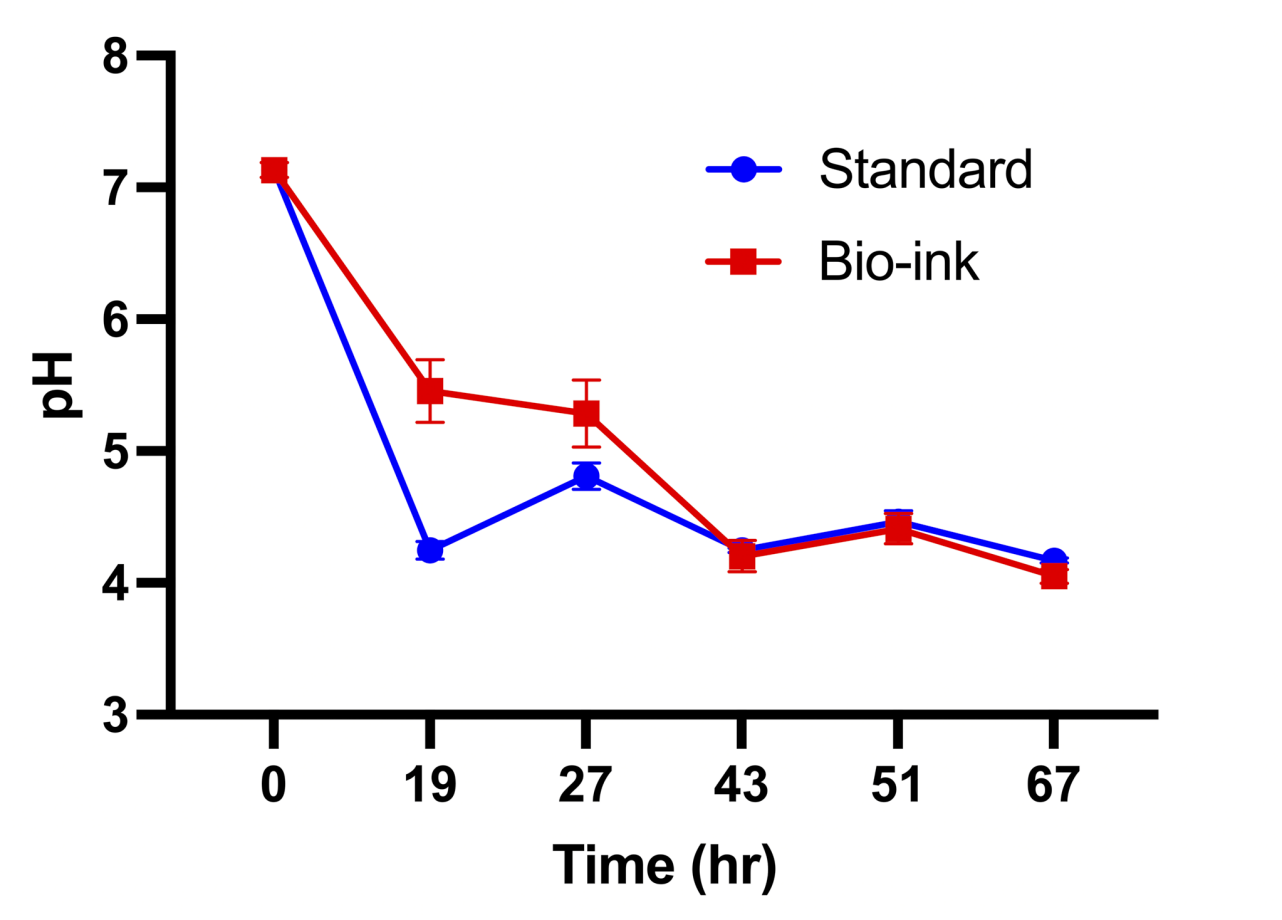
3D-printed oral biofilms acidify their environment. Environmental pH of bio-ink and standard biofilms over time. Mean and standard deviation are plotted for each time point.

Acidification of the environmental pH is an indicator that microorganisms in a cariogenic oral biofilm can interact with the sugar from the culture medium, metabolizing it via pyruvate to produce energy sources to survive. The resultant lactic acid deposits can lead to hydroxyapatite demineralization *in vivo* and bacterial selectivity (15, 16, 20). Bio-ink biofilms exhibited a slower reduction in environmental pH versus standard biofilms at 19 and 27 h (Fig. 4). However, at 43 h and throughout the remainder of the experiment, both the bio-ink and the standard biofilms exhibited pH ~4.5, consistent with previous studies of model *S. mutans* biofilms (23) (Fig. 4). The control biofilms showed pH values that were similar to those of the standard biofilms (Fig. 4; Fig. S3B), indicating that calcium ion supplementation to the bio-ink culture medium and the sodium citrate post-treatment did not interfere with the environmental acidification.

The slower initial drop in pH for the bio-printed biofilms was likely related to the distribution of microorganisms since the microorganisms in the hydrogel were immersed within the sterile culture medium, diluting the acid produced by the bio-printed hydrogel. In comparison, the standard biofilms were immersed within the bacterial culture, as highlighted in Fig. 1, in which all microorganisms were producing acid, including the microbes that were not attached to the hydroxyapatite disk. Therefore, for the 19 h time point, any acid that was created by the bio-ink bacteria was diluted into the clean culture medium, whereas for the standard biofilms, the planktonic bacteria as well as the developing biofilm bacteria were all able to contribute to lowering the pH. Equivalent pH levels were observed for bio-printed and standard biofilms following the first change of the culture medium, which removed free bacteria cells present in the medium for the standard biofilms that did not participate in the biofilm formation process. Thus, starting at the 27 h time point, any acid was produced only by bacteria present in the bio-ink or adherent to the hydroxyapatite surface. These data confirm that the updated 3D-bioprinting methodology was able to allow nutrient and metabolism waste product exchange with the environment as well as to protect the embedded microbes and promote a suitable environment for oral biofilm formation *in vitro*.

### 3D-printed *S. mutans* form patterned oral biofilms

Confocal microscopy was used to track the growth and distribution of *S. mutans* bacteria within the biofilms at 19 h and 67 h. Samples were incubated in the presence of fluorophore-labeled dextran added to the culture medium during biofilm development to label the exopolysaccharides in the extracellular matrix. Prior to imaging, bacterial cells were labeled using SYTO 9, a fluorescent nucleic acid stain. Since the biofilm samples were too thick to allow for confocal microscopy imaging throughout the entire volume of the samples, the microscopy images are provided to illustrate the patterns of spatial organization for the microcolonies and EPS, rather than to show the overall content of these components.

**Fig 5:**
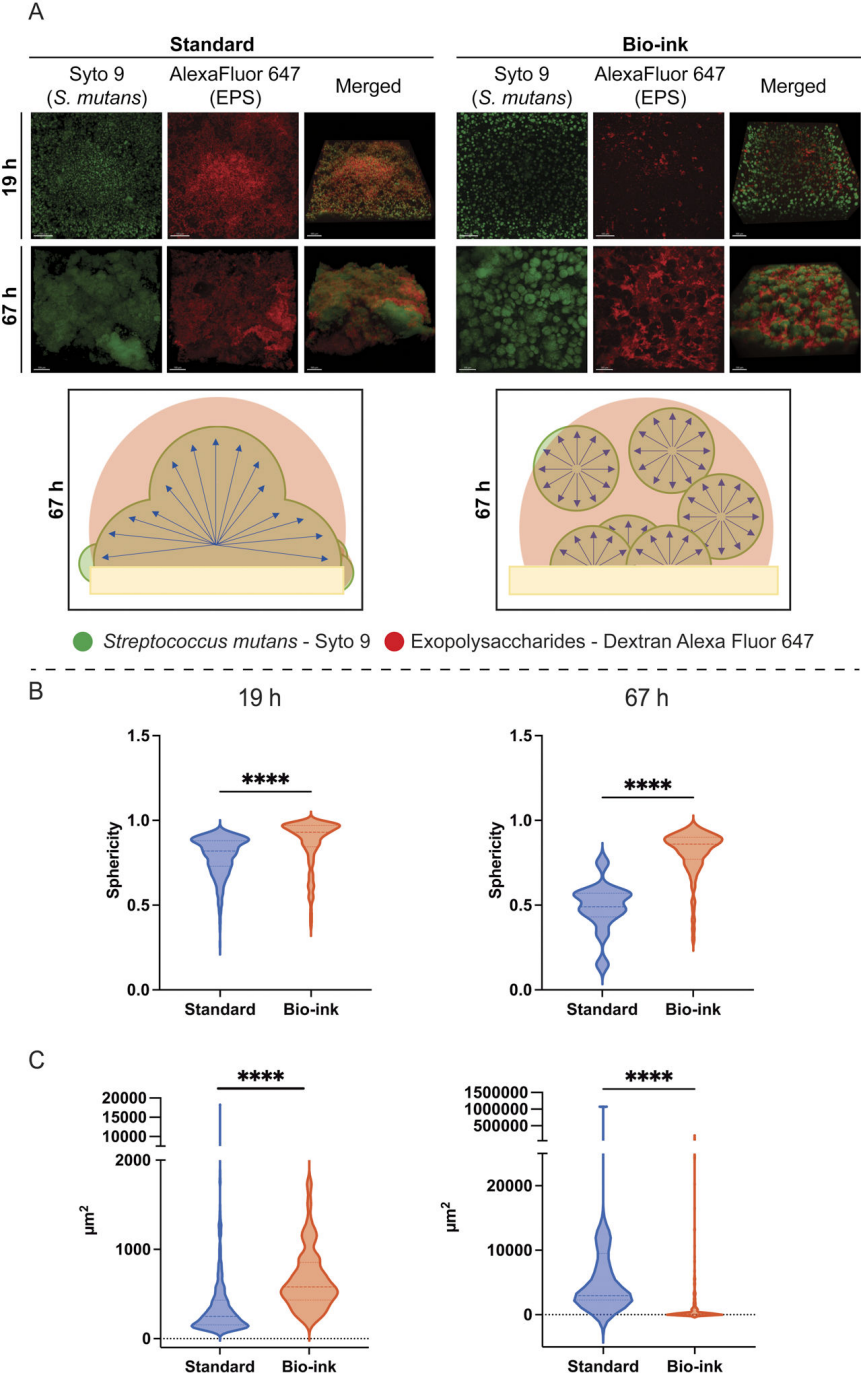
Three-dimen*s*ional oral biofilm structure over time. (**A**) Representative fluorescence confocal microscopy images of 19 h and 67 h *S*. *mutans* biofilms. The green color indicates microorganisms (labeled with SYTO9). The red color represents exopolysaccharides in the extracellular matrix produced by *S. mutans* (labeled with dextran-Alexa Fluor 647). The schematic illustrations show the spatial organization of the bacterial microcolonies (brown) relative to the HA surface (beige) in the standard and the bio-ink biofilms, as seen from the confocal microscopy imaging. Single-channel images are top views, and merged images were tilted at 45°, with the HA surface located at Z = 0 for each of the images. Imaging was performed at 20×. Scale bars represent 100 µm. (**B**) Sphericity of individual microcolonies at 19 h and 67 h, and (C) Surface area (µm^2^) of individual microcolonies at 19 h and 67 h. Quantifications were performed using ImarisViewer 10.1.0. Statistical analyses were performed using a Kruskal-Wallis test, followed by Dunn’s test. **** indicates *P* ≤ 0.0001.

The standard biofilm samples at 19 h were composed of bacterial microcolonies of heterogeneous sizes and shapes with a non-uniform spatial distribution, localized exclusively to the surface of the hydroxyapatite (Fig. 5A). At the same time point, the bio-ink biofilms showed a fairly uniform spatial distribution of microcolonies throughout the height of the sample (Fig. 5A). The bio-ink microcolonies were spatially separated from neighboring colonies and surrounded by the deposited extracellular matrix (Fig. 5A). Quantification of the relative sphericity of the microcolonies revealed that the microcolonies of the bio-ink biofilms were statistically significantly more spherical than those of the standard biofilms (Fig. 5B), and analysis of the microcolony areas indicated that the bio-ink biofilm microcolonies had significantly larger average surface areas (Fig. 5C) at 19 h.

At 67 h, the *S. mutans* microcolonies of the standard biofilms had grown and merged into large cluster-like macrocolonies that were fully embedded within exopolysaccharides and remained localized to the hydroxyapatite surface (Fig. 5A), as described previously in the literature for both standard *in vitro* biofilms (11, 20, 52) and *in vivo* oral biofilms (12, 17, 22, 49, 55). The microcolonies of the 67 h bio-ink biofilms maintained their initial nearly spherical morphologies (Fig. 5B) and had increased radially in size compared with the early time point (Fig. 5A and C). The spatially distributed microbial colonies in the bio-ink biofilms were separated by large areas of exopolysaccharides distributed throughout the entire sample, in contrast to the standard biofilm exopolysaccharide that was largely blanketed overtop of the microbes (Fig. 5A). The quantification indicated that the sphericity of the bio-ink biofilms remained high and similar to the initial time point, in contrast to the standard biofilm microcolonies, which exhibited greatly decreased sphericity and were significantly less spherical than the bio-ink microcolonies at 67 h (Fig. 5B). Microcolony surface area analysis showed that the standard biofilms contained fewer individual microcolonies with an increased average calculated surface area compared to the 19 h time point, while the bio-ink biofilms maintained a higher number of smaller microcolonies, which had also increased in surface area over time and showed significantly smaller average surface areas than the standard biofilm microcolonies (Fig. 5C).

Imaging of the control biofilms revealed that calcium ion supplementation to the bio-ink culture medium and the sodium citrate post-treatment did not contribute to changes in the patterns of microbial or exopolysaccharide distribution in the biofilms (Fig. S4A). These control biofilms all showed a marked decrease in sphericity over time with no statistical difference found between the control groups and the standard biofilms at both 19 and 67 hours (Fig. S4B), as well as coalescence over time of many smaller microcolonies to form larger microcolony clusters, showing no significant differences with the standard biofilms at 67 h (Fig. S4C).

Overall, the confocal microscopy images showed that the bio-ink samples developed oral biofilms on the hydroxyapatite surfaces, containing a rich population of cells living in organized communities highly protected by exopolysaccharides. The microscopy results could potentially indicate changes in biofilm development arising from the bio-ink hydrogel matrix. The initial sparser distribution and greater average surface area of the individual microcolonies in the bio-ink samples may have allowed for the formation of more extensive exopolysaccharide structures surrounding each of the microcolonies, consistent with the larger amount of WSP seen for the bio-ink samples at early time points, and consequently a larger space in which to create matrix deposits compared to the standard method, while also potentially preventing the merging of the microcolonies to form larger units. Further experimentation to observe bio-ink biofilm development over time in concert with different levels of extracellular matrix will be required to determine the mechanisms underlying the spatial distribution of the bacteria.

### 3D-printed oral biofilm structure and mechanical properties mature over early time points

**Fig 6:**
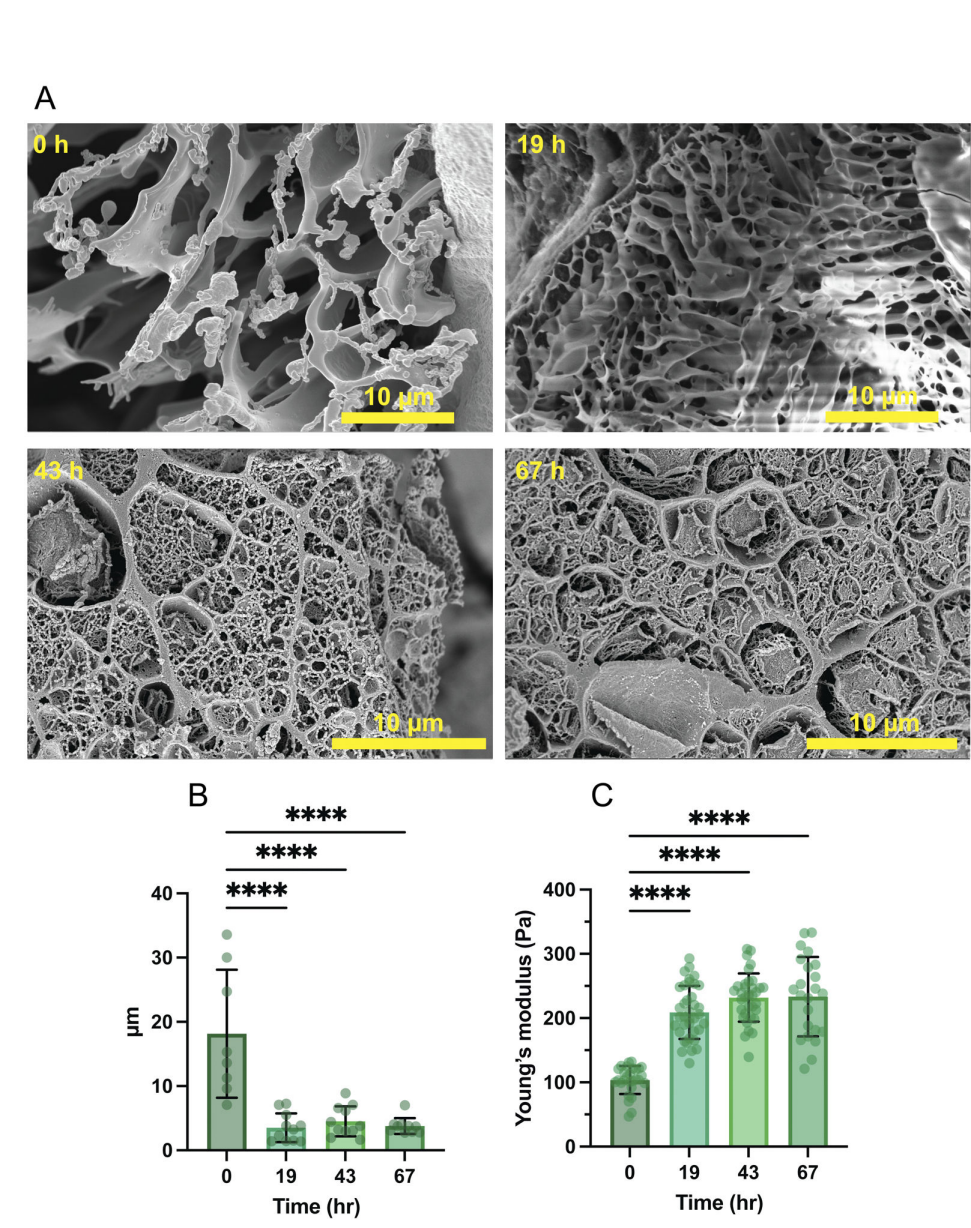
3D-printed oral biofilm structures and mechanical properties mature during early time points. (**A**) Cryo-scanning electron microscopy of bio-ink *S. mutans* biofilms after 0, 19, 43, and 67 h of incubation. (**B**) Hydrogel pore diameter and (**C**) Young’s modulus of bio-ink hydrogels (*n* = 24). Data are shown as mean ± standard deviation using ANOVA with Tukey’s correction for multiple comparisons. **** indicates *P* ≤ 0.0001.

To investigate the effect of biofilm development on the mechanical and structural properties of the bio-ink biofilms, imaging and rheological characterization was performed. Hydrogel structure and pore size of the developing bio-ink biofilms after incubation for varying time points was analyzed by preparing thin cryosections of the biofilms and analyzing them using cryo-scanning electron microscopy (cryo-SEM). The cryo-SEM images revealed the random hydrogel network formed by alginate around the bacteria cells at all time points (Fig. 6A), in agreement with the confocal microscopy images (Fig. 5A). The average pore diameter was approximately 20 µm at the 0 h time point, after which it showed a statistically significant decrease in size, decreasing to an average of approximately 3.5 µm after 19 h. The pore diameter did not show significant changes in size for the 43 or 67 h time points (Fig. 6B). These µm-scale pores are large enough to enable facile transport of water and gas molecules, sucrose, and bacterial byproducts including lactic acid. However, the presence of the hydrogel was not associated with spatial organization of the bacteria into a biofilm in the same way as in the standard biofilms. These cryo-SEM data indicate that the bio-ink hydrogel may have served as a physical barrier to bacterial organization over time, resulting in both increased exopolysaccharide deposition (Fig. 3) and smaller, more spherical isolated microcolonies observed by confocal microscopy at 67 h (Fig. 5). Our bio-ink biofilm model thereby differs from the standard *in vitro* model that currently is the most accepted to mimic the *in vivo* findings, wherein the urbanization sequence results in (i) restriction of biofilm microcolonies to the hydroxyapatite surface and (ii) the combination of smaller microcolonies to become larger clusters within biofilms over time (56).

To analyze the viscoelastic properties of the bio-ink biofilms over time, strain and frequency sweep rheology testing were performed on bio-ink biofilms at different time points to characterize and quantify the linear viscoelastic region (LVR). Strain sweep analysis of the bio-ink biofilms showed a 5% decrease in storage modulus at 0.25% strain (Fig. S5A), which was used as the reference for the following tests. Frequency sweep experiments showed that hydrogels at 0 h displayed a lower storage modulus at frequencies above 10 Hz, compared to the 19 h and later time points, which exhibited a similar, higher storage modulus at >10 Hz frequencies. (Fig. S5B). Throughout the measurements, storage moduli were observed to be greater than loss moduli, indicating predominantly elastic behavior of the bio-ink biofilms. Additional rheological testing was performed to measure the time that it takes the bio-ink biofilms to fully gelate upon the addition of calcium chloride as a crosslinking agent. The hydrogel gelation was observed to begin immediately upon the application of calcium chloride, as seen by an increase in the storage modulus. The gelation process was mostly complete within the first 15–20 seconds, with a slower increase in storage modulus seen over time thereafter (Fig. S5C).

The mechanical stiffness of the bio-ink biofilms over time was measured by performing compression tests. The Young’s modulus showed a significant, nearly twofold increase between the 0 and 19 h time points, after which no statistical differences in hydrogel stiffness were observed (Fig. 6C). Overall, the first 19 h of bio-ink biofilm development were seen to coincide with a decrease in the hydrogel pore size, and this denser matrix displayed higher stiffness and higher ability to store energy elastically while maintaining overall elastic behavior. These mechanical changes in the early period of bio-ink biofilm development could be due to more highly crosslinked hydrogel matrices over time, attributable to a combination of ongoing alginate crosslinking and initial bacterial production of EPS polymers.

Our experiments showed that bio-printed *Streptococcus mutans* was able to proliferate post-deposition, creating biofilms that displayed similarly sized microbial populations, reproduced the standard hallmarks of virulence for oral biofilm virulence factors including biomass and environmental acidification. The bio-ink biofilms displayed increased glucan content compared to standard methods of biofilm formation, which could be a positive factor for research that is focused on evaluating topical treatments by increasing the challenge for the tested therapy via increased exopolysaccharide challenge for the topical treatment. Cryo-SEM and rheology data indicated that the overall structure of the deposited hydrogel was preserved during the development of the bio-ink biofilms and supplemented with bacteria-produced extracellular matrix. This hydrogel network likely allowed the bacteria to grow and divide over time to form microcolonies but partially restricted the developing microcolonies from merging with neighboring microcolonies, as has been seen for other 3D-bioprinted and hydrogel-embedded microbes, impacting the biofilm self-organization (57–59). Future work can also focus on optimizing the 3D-printed hydrogel structure to allow the bacteria to associate in larger clusters, which would mimic the spatial distribution seen in both the standard *in vitro* models and *in vivo* samples. The differences seen in the microcolony spatial distribution may indicate that our bio-ink methodology currently has limited clinical relevance; hydrogel optimization will be crucial for the future success of this 3D-bioprinted *in vitro* technique. Further tests must be performed to determine whether the differences in the three-dimensional microbial distribution will have an impact on biofilm performance in *in vitro* studies such as drug screening or drug efficiency testing (17, 49, 55). After these initial challenges are overcome, we envision that further proof of concept can be addressed by testing this new methodology with several other combinations of methods, including using a multispecies biofilm, modifications to the culture medium by adding saliva or starch, or introducing the bio-ink biofilms into a bioreactor to better mimic the salivary flow and create a dynamic environment. It is early to directly compare this methodology with the *in vivo* environment due to the numerous challenges faced in translating from a developing *in vitro* method to the dynamic *in vivo* oral microbiome.

Following further optimization of the bio-ink hydrogel chemistry, this 3D-bioprinting approach can potentially be the first step for *in vitro* methodologies in several research fields related to oral or general health. Some future directions of this new methodology for 3D-bioprinting oral biofilms can include studying the deposition of extracellular matrix components under external aggressors such as topical treatments, salivary components, or natural/synthetic drugs. This 3D bioprinting technique also opens the possibility to automate oral biofilm formation process *in vitro* without substrate restrictions or with top-down control over the physical dimensions of the biofilm. For future improvements, 3D bioprinting could be combined with bioreactor culturing, allowing greater control over environmental factors such as external pH, sugar availability, and fluid flow, or allowing for the addition of late microbial colonizers over time to study bacterial interactions. Additionally, development of 3D-bioprinted oral biofilms that include incubation with saliva and dietary carbohydrates would allow for closer emulation of *in vivo* parameters affecting biofilm development and adoption of cariogenic phenotypes (21). This 3D bioprinting methodology can also enable deeper characterization of single- and/or multispecies biofilms and can allow the development of new approaches to manage oral biofilms for maintaining and improving health.

## MATERIALS AND METHODS

All chemicals were supplied by Sigma-Aldrich unless stated otherwise. Type 1 MilliQ water with resistivity of 18.2 MΩ.cm or greater (Milli-Q Benchtop Lab Water Purification Systems, Sigma-Aldrich) was used for all solution preparation in this study. All incubation steps were performed at 37°C and 5% CO_2_ (Binder C, Binder Inc., Bohemia, NY). The centrifuge used was Eppendorf 5810R (Eppendorf SE, Hamburg, Germany). The data were organized using Microsoft Excel (Microsoft Corp., Redmond, WA), and the statistical analyses were performed on GraphPad Prism 10 (GraphPad Software, Inc., La Jolla, CA). The software recognized no statistical outliers to be removed from the tests. One-way ANOVA or Kruskal-Wallis tests were performed, employing a significance level fixed at 5%, followed by *post hoc* Tukey’s or Dunn’s to perform multi-comparison tests. The statistical tests were performed to compare groups tested within specific time points, and the specific statistical method used was based on the Shapiro-Wilk test result.

### Culture medium

The culture medium used to create the bio-ink was 2.5% tryptone, 1.5% yeast extract, and 1.5% alginate, supplemented by 1% sucrose. The alginate was added during the medium preparation before autoclaving to prevent dilution of the culture medium with alginate. The standard culture medium used to submerge the bio-ink and perform media changes was 2.5% tryptone, 1.5% yeast extract, supplemented by 1% sucrose and 0.1 M calcium chloride. Since the culture medium was an isotonic solution that could dissolve the alginate hydrogel after several hours, 0.1 M calcium chloride was added to the bio-ink and standard + calcium group as a culture medium supplementation to stabilize the hydrogel for longer incubation periods and as a control for our experiments. The supplementation was present on all culture medium changes. The control groups contained or lacked the calcium chloride supplementation depending on the experimental design.

Upon reaching the age required for data collection, some of the samples were submerged into a solution of 0.5 M sodium citrate for 2 h after the experimental period (43 h) to remove the alginate following the experimental group design. The culture medium was changed twice daily at 19, 27, 43, and 51 h.

### Experimental group design

Experimental groups were created to verify biofilm formation using this novel bio-ink methodology versus traditional accumulation on the mineral surface. The experimental design goals were to observe the impact of calcium supplementation in the culture medium and the impact of post-treatment using sodium citrate to depolymerize the bio-ink. Thus, the groups were divided into (i) standard (standard growth without calcium chloride and no sodium citrate treatment), (ii) standard + sodium citrate (standard growth with calcium chloride supplementation and sodium citrate treatment), (iii) standard + calcium (standard growth with calcium chloride supplementation and no sodium citrate treatment), and (iv) bio-ink (calcium chloride supplementation and sodium citrate treatment). The culture medium was changed at 0, 19, 27, 43, and 51 h (10, 13, 23, 36, 49). The biofilm was incubated undisturbed until the first change of culture media at the 19 h time point to allow bacterial adhesion to the hydroxyapatite surface in the initial stages of biofilm formation. The culture medium was changed twice a day thereafter until the end of the experiment to provide fresh nutrients to the biofilm. The pH measurements (VWR Symphony B10P) were made immediately after media changes, including at the end of experiment (43 or 67 h).

### Substrate preparation

Hydroxyapatite disks (HA) were purchased from Clarkson Chromatography Co. with size 0.25 in. diameter × 0.008 in. thick. The disks were autoclaved, stored at room temperature, and hydrated in MilliQ water for 30 min. Disks were then transferred to 0.1 M calcium chloride solution for 10 min. Excess calcium was removed using a pipette tip before deposition of bio-ink on the surface or contact with culture medium.

### Bacterial strain and culture

**Fig 7:**
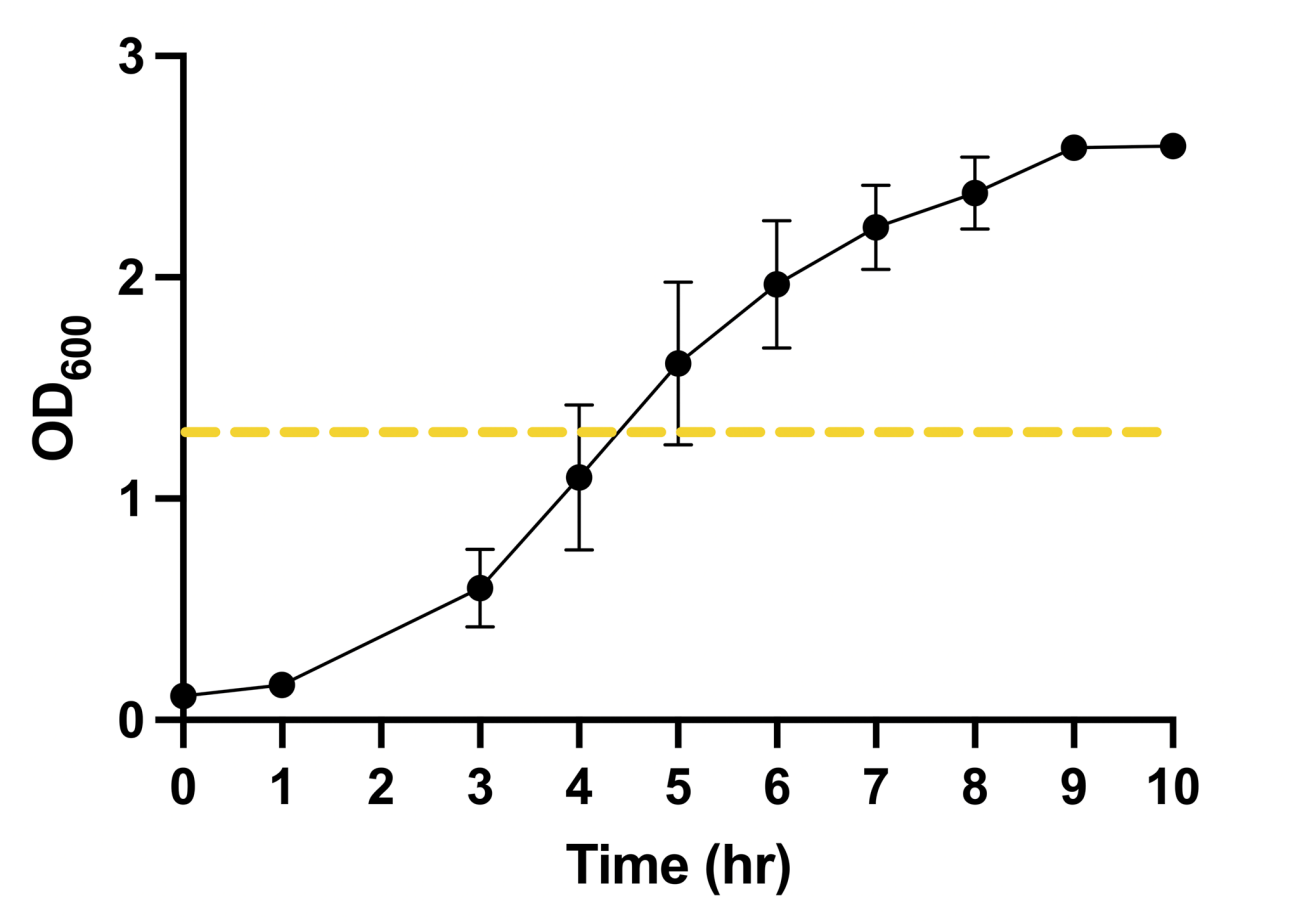
Standard growth curve of *Streptococcus mutans* UA159 measured using a spectrophotometer. The inoculum used on all experiments was prepared when the bacteria achieved mid-log of the exponential phase, highlighted with an orange dotted line. The concentration of microorganisms was confirmed via a spectrophotometer for all experiments as a control.

*Streptococcus mutans* UA159 (ATCC 700610) was used for all experiments. The strain was stored at −80°C in 2× TYE (concentrated tryptone with yeast extract) with 20% glycerol as a cryoprotectant. Frozen stocks were seeded onto blood agar plates for 48 h. After this period, five to ten colonies were inoculated into 10 mL of culture medium 2.5% tryptone, 1.5% yeast extract supplemented by 1% glucose (TYE + 1% glucose) for 16 h of incubation in duplicate. Then, 0.5 mL of the pre-inoculum was diluted at 1:20 (vol/vol) into new 15 mL tubes with fresh TYE + 1% glucose. The *S. mutans* cultures were grown until the mid-log growth phase, based on a previously developed standard curve (O.D._600_ 1.24 ± 0.341) determined with a NanoDrop One/OneC Microvolume UV-Vis Spectrophotometer (Fig. 7). Finally, the final inoculums were prepared by mixing an aliquot of mid-log phase culture at the correct O.D._600_ into 15 mL of the fresh culture medium to obtain the final 10^7^ CFU/mL; aliquot volumes were determined from the bacterial standard curve measured for the Meyer laboratory and instrumentation. Final inoculums were prepared in two different vials: (i) TYE supplemented with 1% sucrose for control groups that used standard methodology and (ii) TYE supplemented with 1% sucrose and 1.5% sodium alginate (wt/vol) for bio-ink samples. As a verification step for experimental normalization, extra volume of the inoculum was prepared to ensure that 100 µL could be collected for serial dilution and culturing on blood agar plates, in order to confirm that the initial inoculum was prepared at 10^7^ CFU/mL.

Experiments were performed in duplicates and repeated three times. For the bio-ink preparation, two HA disks were placed in each well of a 24-well plate, excess calcium solution was removed using a pipette, then 30 µL of inoculum followed by 10 µL of 0.1 M calcium chloride were deposited onto the top of the HA disks, followed by a 5 min incubation to allow alginate gelation. Groups 2, 3, and 4 were incubated in culture medium supplemented with 0.1 M calcium chloride, and control group 1 was submerged directly into the inoculum without calcium supplementation.

### Biofilm analysis

** **Biofilms from groups 2 and 4 were carefully dip-washed three times in saline solution (0.89% NaCl), transferred to a 0.5 M sodium citrate solution, and continuously shaken at 45 rpm at room temperature for 2 h for alginate depolymerization, after which they followed the same collection steps. To collect the biofilm from the HA surface, the disks were placed into saline solution, and the biofilm was completely removed from the surface using autoclaved stainless-steel spatulas. The procedure was performed twice for each disk to collect as much biofilm as possible from the surface. The well and the disk were then rinsed twice. The final volume used was 5 mL of saline per disk. The suspension was collected in new 15 mL tubes. The biofilm was then probe sonicated at 7–10 watts for 30 s (Branson Ultrasonic Sonifier Power 450) to homogenize the bacterial suspension. The sample was diluted into 100 µL aliquots to prepare 10-fold serial dilutions, which were seeded onto blood agar plates to determine CFU/mL. The agar plates were incubated for 48 h, followed by manual enumeration of colonies. The remaining suspension (4.9 mL) was centrifuged at 4,000 rpm for 20 min at 4°C. The supernatant was collected in a 50 mL tube. Each pellet was washed two more times with 2.5 mL MilliQ water. The supernatant was stored at −20°C and used to analyze water-soluble polysaccharides (WSP), following the extraction process described below. The pellets were suspended in 2.5 mL of MilliQ water, after which 0.5 mL was used to analyze insoluble dry weight (biomass), and 1 mL was used to analyze alkali-soluble polysaccharides (ASP).

 Pre-weighed weighing boats made of aluminum foil were used to contain the 0.5 mL aliquots. The boats were placed in an oven at 90°C overnight. The difference between the initial and final mass provided the insoluble dry weight values. The pre-weighed centrifuge tubes containing the 1 mL aliquot of ASP were centrifuged at 14,000 rpm for 10 min at 4°C, and the supernatant was carefully removed. Then, 0.3 mL of 0.5 M NaOH per 1 mg of wet biofilm weight was added to the sample. All samples were continuously shaken at 90 rpm for 2 h at 37°C, followed by centrifugation at 14,000 rpm for 10 min at 4°C, and then the supernatant was collected into new 15 mL tubes. This cycle of extraction was performed three times before discarding the pellet.

The tubes containing the WSP and ASP extraction samples were diluted 1:3 (vol/vol) using 95% ethanol. The samples were stored at −20°C for precipitation for at least 18 h, followed by centrifugation at 4,000 rpm for 20 min at 4°C, followed by three washes with 75% ethanol and left to dry for 30 min at room temperature. WPS samples were dissolved in 1 mL of autoclaved MilliQ water, and the ASP samples were dissolved in 0.3 mL of 0.5 M NaOH per 1 mg. Quantification of WSP and ASP was performed using a phenol-sulfuric acid colorimetric assay with glucose as a standard (54).

### Confocal fluorescence microscopy

 For confocal fluorescence microscopy, biofilms were grown following the same regimen described above, with the addition of 1 µM Alexa Fluor 647 conjugated to dextran (ThermoFisher D22914) mixed into the culture medium to label the exopolysaccharides in the matrix. The biofilms were rinsed using sterilized saline solution and transferred to wells containing 2.6 µM SYTO 9 (ThermoFisher S34854) in saline solution for 30 min to label the bacterial nucleic acids (26). The samples were washed in three different and unique wells containing autoclaved MilliQ water. The samples were placed into fresh saline solution until imaging with an Andor Dragonfly Spinning Disc Confocal, Oxford Instruments. Fusion software was used to acquire a minimum of three images per sample at two different magnifications (20× and 40× objectives). ImarisViewer software (Oxford Instruments, version 10.1.0) was used to process the final images and calculate the biofilm area and microcolony sphericity.

To calculate the sphericity, the software first rendered the microcolony image into a surface by reconstructing it using a 3D mesh made of triangles. The surface area was calculated as the sum of all triangle facet areas that make up the 3D mesh of the microcolony surface. Sphericity was calculated as the ratio of the surface area of a sphere with the same volume as each sample to the surface area of the corresponding sample (60). The values vary from 0, indicating a complete lack of sphericity, to 1, indicating perfect sphericity.

### Cryo-scanning electron microscopy

 Biofilm samples were created by pipetting 30 µL of *S. mutans* UA159 culture supplemented with 1% sucrose and 1.5% alginate onto HA disks, followed by the addition of 10 µL of 0.1 M calcium chloride overtop. The sample was placed into culture medium supplemented with 0.1 M calcium chloride (as in Fig. 1A). Samples were incubated for 0, 19, 43, and 67 h at 37°C and 5% CO_2_. The samples were transferred to a metal SEM plate. The samples were frozen using liquid nitrogen for 5 min under vacuum and then transferred under vacuum into the cryo-chamber. Samples were coated with platinum under argon for 4 min and were carefully sliced using a sharp scalpel in order to observe the cross-section of the freeze-dried biofilms. The coated samples were transferred to the microscope main chamber under vacuum and imaged at 5 kV and 0.69 nA (FEI Helios DualBeam FIB 600, ThermoFisher, US). ImageJ software (National Institutes of Health-NIH and the Laboratory for Optical and Computational Instrumentation-LOCI, University of Wisconsin, US, version 1.53t) was used to process the final images and calculate hydrogel pore size.

### Rheology testing

Biofilm samples were prepared in a 24-well plate using TYE + 1% sucrose + 1.5% alginate to which 0.1 M calcium chloride was added in a 3:1 ratio (vol/vol). Samples were prepared on site for gelation time testing and viscoelastic testing at the 0 h time points. For all Young’s modulus testing samples and for incubated samples for gelation time testing and viscoelastic testing, samples were covered by 2 mL of culture medium supplemented with 0.1 M calcium chloride and incubated at 37°C and 5% CO_2_ for 19, 43, and 67 h. A biopsy punch was used to obtain 8 × 3 mm samples of the samples.

Viscoelastic characterization of the samples was performed on a TA Instruments RSA-G2 system, which provided a quantification of the linear viscoelastic region (LVR). Testing was performed at room temperature (21°C) using a set of 15 mm compression platens. Strain sweeps were performed between 0.01% and 10% strain at 1 Hz. The reference strain was determined by 5% change in storage modulus (0.25% strain). Frequency sweeps were performed from 0.1 to 100 Hz at 0.25% strain (48).

Gelation time testing was performed on a TA Instruments Discovery HR 30 system. Bacteria culture with 1.5% alginate was deposited on site, making sure the sample was in contact with both 8 mm plates. Analysis was performed with a gap of 2,500 µm, trim gap offset 125 µm, and duration 1,800 s, using 0.25% strain at 1 Hz. The 0.1M calcium chloride was added exactly 60 seconds after the start of the gelation time data collection.

The Young’s modulus of the biofilm samples was characterized by performing compression testing using a TA Instruments RSA-G2 system. An 8 mm plate was used, with a constant linear rate of 0.05 mm/s, and the samples were compressed until failure. At least nine points were selected to extract the equation for a straight line on the linear region of the data. The calculated *R*^2^ needed to be greater than 0.98 for each sample to be considered on our test. The Young’s modulus was calculated via the area under the initial section of the linear curve identified at the previous step.

## SUPPLEMENTAL MATERIAL

Supplemental figures spectrum.00935-25-s0001.docx Fig. S1 to S5.

ASM does not own the copyrights to Supplemental Material that may be linked to, or accessed through, an article. The authors have granted ASM a non-exclusive, world-wide license to publish the Supplemental Material files. Please contact the corresponding author directly for reuse.

## References

[1] Branda SS, Vik S, Friedman L, Kolter R. 2005. Biofilms: the matrix revisited. Trends Microbiol 13:20–26. doi:10.1016/j.tim.2004.11.006 15639628

[2] Selwitz RH, Ismail AI, Pitts NB. 2007. Dental caries. Lancet 369:51–59. doi:10.1016/S0140-6736(07)60031-2 17208642

[3] Xiao J, Hara AT, Kim D, Zero DT, Koo H, Hwang G. 2017. Biofilm three-dimensional architecture influences in situ pH distribution pattern on the human enamel surface. Int J Oral Sci 9:74–79. doi:10.1038/ijos.2017.8 28452377 PMC5518976

[4] Vacca-Smith AM, Bowen WH. 1998. Binding properties of streptococcal glucosyltransferases for hydroxyapatite, saliva-coated hydroxyapatite, and bacterial surfaces. Arch Oral Biol 43:103–110. doi:10.1016/s0003-9969(97)00111-8 9602288

[5] Takahashi N, Nyvad B. 2011. The role of bacteria in the caries process. J Dent Res 90:294–303. doi:10.1177/0022034510379602 20924061

[6] Hajishengallis E, Parsaei Y, Klein MI, Koo H. 2017. Advances in the microbial etiology and pathogenesis of early childhood caries. Mol Oral Microbiol 32:24–34. doi:10.1111/omi.12152 26714612 PMC4929038

[7] Benzian H, Watt R, Makino Y, Stauf N, Varenne B. 2022. WHO calls to end the global crisis of oral health. Lancet 400:1909–1910. doi:10.1016/S0140-6736(22)02322-4 36410360

[8] Kidd EAM, Fejerskov O. 2004. What constitutes dental caries? Histopathology of carious enamel and dentin related to the action of cariogenic biofilms. J Dent Res 83:35–38. doi:10.1177/154405910408301s07 15286119

[9] Klein MI, Duarte S, Xiao J, Mitra S, Foster TH, Koo H. 2009. Structural and molecular basis of the role of starch and sucrose in Streptococcus mutans biofilm development. Appl Environ Microbiol 75:837–841. doi:10.1128/AEM.01299-08 19028906 PMC2632160

[10] Klein MI, Xiao J, Lu B, Delahunty CM, Yates JR, Koo H. 2012. Streptococcus mutans protein synthesis during mixed-species biofilm development by high-throughput quantitative proteomics. PLoS One 7:e45795. doi:10.1371/journal.pone.0045795 23049864 PMC3458072

[11] Xiao J, Klein MI, Falsetta ML, Lu B, Delahunty CM, Yates JR, Heydorn A, Koo H. 2012. The exopolysaccharide matrix modulates the interaction between 3D architecture and virulence of a mixed-species oral biofilm. PLoS Pathog 8:e1002623. doi:10.1371/journal.ppat.1002623 22496649 PMC3320608

[12] Bowen WH, Burne RA, Wu H, Koo H. 2018. Oral biofilms: pathogens, matrix, and polymicrobial interactions in microenvironments. Trends Microbiol 26:229–242. doi:10.1016/j.tim.2017.09.008 29097091 PMC5834367

[13] Lemos JA, Abranches J, Koo H, Marquis RE, Burne RA. 2010. Protocols to study the physiology of oral biofilms, p 87–102. Humana Press. 10.1007/978-1-60761-820-1_7 PMC3130507 20717780

[14] Castillo Pedraza MC, Novais TF, Faustoferri RC, Quivey RG, Terekhov A, Hamaker BR, Klein MI. 2017. Extracellular DNA and lipoteichoic acids interact with exopolysaccharides in the extracellular matrix of Streptococcus mutans biofilms. Biofouling 33:722–740. doi:10.1080/08927014.2017.1361412 28946780 PMC5929139

[15] Lemos JA, Palmer SR, Zeng L, Wen ZT, Kajfasz JK, Freires IA, Abranches J, Brady LJ. 2019. The biology of Streptococcus mutans. Microbiol Spectr 7. doi:10.1128/microbiolspec.GPP3-0051-2018 PMC6615571 30657107

[16] Lamont RJ, Koo H, Hajishengallis G. 2018. The oral microbiota: dynamic communities and host interactions. Nat Rev Microbiol 16:745–759. doi:10.1038/s41579-018-0089-x 30301974 PMC6278837

[17] Costa RC, Bertolini M, Costa Oliveira BE, Nagay BE, Dini C, Benso B, Klein MI, Barāo VAR, Souza JGS. 2023. Polymicrobial biofilms related to dental implant diseases: unravelling the critical role of extracellular biofilm matrix. Crit Rev Microbiol 49:370–390. doi:10.1080/1040841X.2022.2062219 35584310

[18] He J, Hwang G, Liu Y, Gao L, Kilpatrick-Liverman L, Santarpia P, Zhou X, Koo H. 2016. L-Arginine modifies the exopolysaccharide matrix and thwarts Streptococcus mutans outgrowth within mixed-species oral biofilms. J Bacteriol 198:2651–2661. doi:10.1128/JB.00021-16 27161116 PMC5019072

[19] Cheng X, Redanz S, Cullin N, Zhou X, Xu X, Joshi V, Koley D, Merritt J, Kreth J. 2018. Plasticity of the pyruvate node modulates hydrogen peroxide production and acid tolerance in multiple oral streptococci. Appl Environ Microbiol 84:e01697-17. doi:10.1128/AEM.01697-17 29079629 PMC5752870

[20] Kim D, Barraza JP, Arthur RA, Hara A, Lewis K, Liu Y, Scisci EL, Hajishengallis E, Whiteley M, Koo H. 2020. Spatial mapping of polymicrobial communities reveals a precise biogeography associated with human dental caries. Proc Natl Acad Sci USA 117:12375–12386. doi:10.1073/pnas.1919099117 32424080 PMC7275741

[21] Florez Salamanca EJ, Dantas RM, Rodriguez MJ, Klein MI. 2020. Establishment of microcosm biofilm models that reproduce a cariogenic diet intake. Biofouling 36:1196–1209. doi:10.1080/08927014.2020.1862093 33349045

[22] Karygianni L, Ren Z, Koo H, Thurnheer T. 2020. Biofilm matrixome: extracellular components in structured microbial communities. Trends Microbiol 28:668–681. doi:10.1016/j.tim.2020.03.016 32663461

[23] Roncari Rocha G, Sims KR Jr, Xiao B, Klein MI, Benoit DSW. 2022. Nanoparticle carrier co-delivery of complementary antibiofilm drugs abrogates dual species cariogenic biofilm formation in vitro. J Oral Microbiol 14:1997230. doi:10.1080/20002297.2021.1997230 34868474 PMC8635615

[24] Xiao J, Koo H. 2010. Structural organization and dynamics of exopolysaccharide matrix and microcolonies formation by Streptococcus mutans in biofilms. J Appl Microbiol 108:2103–2113. doi:10.1111/j.1365-2672.2009.04616.x 19941630

[25] Koo H, Falsetta ML, Klein MI. 2013. The exopolysaccharide matrix. J Dent Res 92:1065–1073. doi:10.1177/0022034513504218 24045647 PMC3834652

[26] Klein MI, Hwang G, Santos PHS, Campanella OH, Koo H. 2015. Streptococcus mutans-derived extracellular matrix in cariogenic oral biofilms. Front Cell Infect Microbiol 5:10. doi:10.3389/fcimb.2015.00010 25763359 PMC4327733

[27] Diaz PI, Xie Z, Sobue T, Thompson A, Biyikoglu B, Ricker A, Ikonomou L, Dongari-Bagtzoglou A. 2012. Synergistic interaction between Candida albicans and commensal oral streptococci in a novel in vitro mucosal model. Infect Immun 80:620–632. doi:10.1128/IAI.05896-11 22104105 PMC3264323

[28] Rainey K, Michalek SM, Wen ZT, Wu H. 2019. Glycosyltransferase-mediated biofilm matrix dynamics and virulence of Streptococcus mutans. Appl Environ Microbiol 85:02247–02218. doi:10.1128/AEM.02247-18 PMC6384114 30578260

[29] Koo H, Hayacibara MF, Schobel BD, Cury JA, Rosalen PL, Park YK, Vacca-Smith AM, Bowen WH. 2003. Inhibition of Streptococcus mutans biofilm accumulation and polysaccharide production by apigenin and tt-farnesol. J Antimicrob Chemother 52:782–789. doi:10.1093/jac/dkg449 14563892

[30] van Hijum SAFT, Kralj S, Ozimek LK, Dijkhuizen L, van Geel-Schutten IGH. 2006. Structure-function relationships of glucansucrase and fructansucrase enzymes from lactic acid bacteria. Microbiol Mol Biol Rev 70:157–176. doi:10.1128/MMBR.70.1.157-176.2006 16524921 PMC1393251

[31] Cottier F, Sherrington S, Cockerill S, Del Olmo Toledo V, Kissane S, Tournu H, Orsini L, Palmer GE, Pérez JC, Hall RA. 2019. Remasking of Candida albicans β-glucan in response to environmental pH is regulated by quorum sensing. mBio 10:e02347-19. doi:10.1128/mBio.02347-19 31615961 PMC6794483

[32] Rocha GR, Florez Salamanca EJ, de Barros AL, Lobo CIV, Klein MI. 2018. Effect of tt-farnesol and myricetin on in vitro biofilm formed by Streptococcus mutans and Candida albicans. BMC Complement Altern Med 18:61. doi:10.1186/s12906-018-2132-x 29444673 PMC5813409

[33] Sims KR, Maceren JP, Liu Y, Rocha GR, Koo H, Benoit DSW. 2020. Dual antibacterial drug-loaded nanoparticles synergistically improve treatment of Streptococcus mutans biofilms. Acta Biomater 115:418–431. doi:10.1016/j.actbio.2020.08.032 32853808 PMC7530141

[34] Eidenhardt Z, Ritsert A, Shankar-Subramanian S, Ebel S, Margraf-Stiksrud J, Deinzer R. 2021. Tooth brushing performance in adolescents as compared to the best-practice demonstrated in group prophylaxis programs: an observational study. BMC Oral Health 21:359. doi:10.1186/s12903-021-01692-z 34284767 PMC8290393

[35] Cieplik F, Jakubovics NS, Buchalla W, Maisch T, Hellwig E, Al-Ahmad A. 2019. Resistance toward chlorhexidine in oral bacteria - is there cause for concern? Front Microbiol 10:587. doi:10.3389/fmicb.2019.00587 30967854 PMC6439480

[36] Kim D, Hwang G, Liu Y, Wang Y, Singh AP, Vorsa N, Koo H. 2015. Cranberry flavonoids modulate cariogenic properties of mixed-species biofilm through exopolysaccharides-matrix disruption. PLoS One 10:e0145844. doi:10.1371/journal.pone.0145844 26713438 PMC4699891

[37] Huang J, Liu S, Zhang C, Wang X, Pu J, Ba F, Xue S, Ye H, Zhao T, Li K, Wang Y, Zhang J, Wang L, Fan C, Lu TK, Zhong C. 2019. Programmable and printable Bacillus subtilis biofilms as engineered living materials. Nat Chem Biol 15:34–41. doi:10.1038/s41589-018-0169-2 30510190

[38] Draget KI, Skjåk-Braek G, Smidsrød O. 1997. Alginate based new materials. Int J Biol Macromol 21:47–55. doi:10.1016/s0141-8130(97)00040-8 9283015

[39] Duan B, Hockaday LA, Kang KH, Butcher JT. 2013. 3D bioprinting of heterogeneous aortic valve conduits with alginate/gelatin hydrogels. J Biomed Mater Res A 101:1255–1264. doi:10.1002/jbm.a.34420 23015540 PMC3694360

[40] Murphy SV, Atala A. 2014. 3D bioprinting of tissues and organs. Nat Biotechnol 32:773–785. doi:10.1038/nbt.2958 25093879

[41] Vicini S, Castellano M, Mauri M, Marsano E. 2015. Gelling process for sodium alginate: new technical approach by using calcium rich micro-spheres. Carbohydr Polym 134:767–774. doi:10.1016/j.carbpol.2015.08.064 26428184

[42] Lehner BAE, Schmieden DT, Meyer AS. 2017. A straightforward approach for 3D bacterial printing. ACS Synth Biol 6:1124–1130. doi:10.1021/acssynbio.6b00395 28225616 PMC5525104

[43] Schmieden DT, Basalo Vázquez SJ, Sangüesa H, van der Does M, Idema T, Meyer AS. 2018. Printing of patterned, engineered E. coli biofilms with a low-cost 3D printer. ACS Synth Biol 7:1328–1337. doi:10.1021/acssynbio.7b00424 29690761

[44] Balasubramanian S, Yu K, Cardenas DV, Aubin-Tam M-E, Meyer AS. 2021. Emergent biological endurance depends on extracellular matrix composition of three-dimensionally printed Escherichia coli biofilms. ACS Synth Biol 10:2997–3008. doi:10.1021/acssynbio.1c00290 34652130 PMC8609572

[45] Lazarus E, Meyer AS, Ikuma K, Rivero IV. 2024. Three dimensional printed biofilms: fabrication, design and future biomedical and environmental applications. Microb Biotechnol 17:e14360. doi:10.1111/1751-7915.14360 38041693 PMC10832517

[46] González LM, Mukhitov N, Voigt CA. 2020. Resilient living materials built by printing bacterial spores. Nat Chem Biol 16:126–133. doi:10.1038/s41589-019-0412-5 31792444

[47] Spiesz EM, Yu K, Lehner BAE, Schmieden DT, Aubin-Tam ME, Meyer AS. 2019. Three-dimensional patterning of engineered biofilms with a do-it-yourself bioprinter. J Vis Exp 147. doi:10.3791/59477 31157785

[48] Kloxin AM, Kloxin CJ, Bowman CN, Anseth KS. 2010. Mechanical properties of cellularly responsive hydrogels and their experimental determination. Adv Mater 22:3484–3494. doi:10.1002/adma.200904179 20473984 PMC3890982

[49] Falsetta ML, Klein MI, Colonne PM, Scott-Anne K, Gregoire S, Pai C-H, Gonzalez-Begne M, Watson G, Krysan DJ, Bowen WH, Koo H. 2014. Symbiotic relationship between Streptococcus mutans and Candida albicans synergizes virulence of plaque biofilms in vivo. Infect Immun 82:1968–1981. doi:10.1128/IAI.00087-14 24566629 PMC3993459

[50] Schneider-Rayman M, Steinberg D, Sionov RV, Friedman M, Shalish M. 2021. Effect of epigallocatechin gallate on dental biofilm of Streptococcus mutans: an in vitro study. BMC Oral Health 21:447. doi:10.1186/s12903-021-01798-4 34525984 PMC8444437

[51] Sundarraman D, Smith TJ, Kast JVZ, Guillemin K, Parthasarathy R. 2022. Disaggregation as an interaction mechanism among intestinal bacteria. Biophys J 121:3458–3473. doi:10.1016/j.bpj.2022.08.010 35982615 PMC9515126

[52] Flemming H-C, Wingender J. 2010. The biofilm matrix. Nat Rev Microbiol 8:623–633. doi:10.1038/nrmicro2415 20676145

[53] Wingender J, Strathmann M, Rode A, Leis A, Flemming HC. 2001. Isolation and biochemical characterization of extracellular polymeric substances from Pseudomonas aeruginosa. Methods Enzymol 336:302–314. doi:10.1016/s0076-6879(01)36597-7 11398408

[54] DuBois M, Gilles KA, Hamilton JK, Rebers PA, Smith F. 1956. Colorimetric method for determination of sugars and related substances. Anal Chem 28:350–356. doi:10.1021/ac60111a017

[55] Falsetta ML, Klein MI, Lemos JA, Silva BB, Agidi S, Scott-Anne KK, Koo H. 2012. Novel antibiofilm chemotherapy targets exopolysaccharide synthesis and stress tolerance in Streptococcus mutans to modulate virulence expression in vivo. Antimicrob Agents Chemother 56:6201–6211. doi:10.1128/AAC.01381-12 22985885 PMC3497192

[56] Paula AJ, Hwang G, Koo H. 2020. Dynamics of bacterial population growth in biofilms resemble spatial and structural aspects of urbanization. Nat Commun 11:1354. doi:10.1038/s41467-020-15165-4 32170131 PMC7070081

[57] Riedel F, Bartolomé MP, Enrico LLT, Fink-Straube C, Duong CN, Gherlone F, Huang Y, Valiante V, Del Campo A, Sankaran S. 2023. Engineered living materials for the conversion of a low-cost food-grade precursor to a high-value flavonoid. Front Bioeng Biotechnol 11:1278062. doi:10.3389/fbioe.2023.1278062 38090710 PMC10715425

[58] Bhusari S, Kim J, Polizzi K, Sankaran S, Del Campo A. 2023. Encapsulation of bacteria in bilayer pluronic thin film hydrogels: a safe format for engineered living materials. Biomater Adv 145:213240. doi:10.1016/j.bioadv.2022.213240 36577192

[59] Oh JJ, Ammu S, Vriend VD, Kieffer R, Kleiner FH, Balasubramanian S, Karana E, Masania K, Aubin-Tam MEG. 2024. Growth, distribution, and photosynthesis of Chlamydomonas reinhardtii in 3D hydrogels. Adv Mater 36:e2305505. doi:10.1002/adma.202305505 37851509

[60] Wadell H. 1934. The coefficient of resistance as a function of Reynolds number for solids of various shapes. J Franklin Inst 217:459–490. doi:10.1016/S0016-0032(34)90508-1

