# Supplementary material for: Reproducible 3D bioprinting of Streptococcus mutans to create model oral biofilms
Source: Microbiol Spectr. 2025 Oct 10;13(11):e00935-25. doi: 10.1128/spectrum.00935-25 (PMC12584641; doi:10.1128/spectrum.00935-25)
Supplement: Supplemental figures — Fig. S1 to S5. [file spectrum.00935-25-s0001.docx]

**Reproducible 3D bioprinting of *Streptococcus mutans* to create model oral biofilms**

**Supplementary Figures**

Supplementary Figure S1. Components of the bio-ink culture medium and bio-ink post-treatments do not interfere with bacterial growth. A. Insoluble dry weight of standard biofilms. B. Population of viable *S. mutans* in standard biofilms. Data are shown as mean ± standard deviation from three independent experiments in duplicate using ANOVA with Tukey’s correction for multiple comparisons. ** indicates p ≤ 0.01, * indicates p ≤ 0.05. CFU/mL = colony-forming units per milliliter.

Supplementary Figure S2. Components of the bio-ink culture medium and bio-ink post-treatments do not interfere with exopolysaccharide matrix deposition. A. Water-soluble exopolysaccharides, and B. Alkali-soluble exopolysaccharides of standard *S. mutans* biofilms. Data are shown as mean ± standard deviation from three independent experiments in duplicate using ANOVA with Tukey’s correction for multiple comparisons. * Indicates p ≤ 0.05.

Supplementary Figure S3. Components of the bio-ink culture medium and bio-ink post-treatments do not interfere with acidification of the environment. Environmental pH of standard biofilms over time. Mean and standard deviation are plotted for each time point.


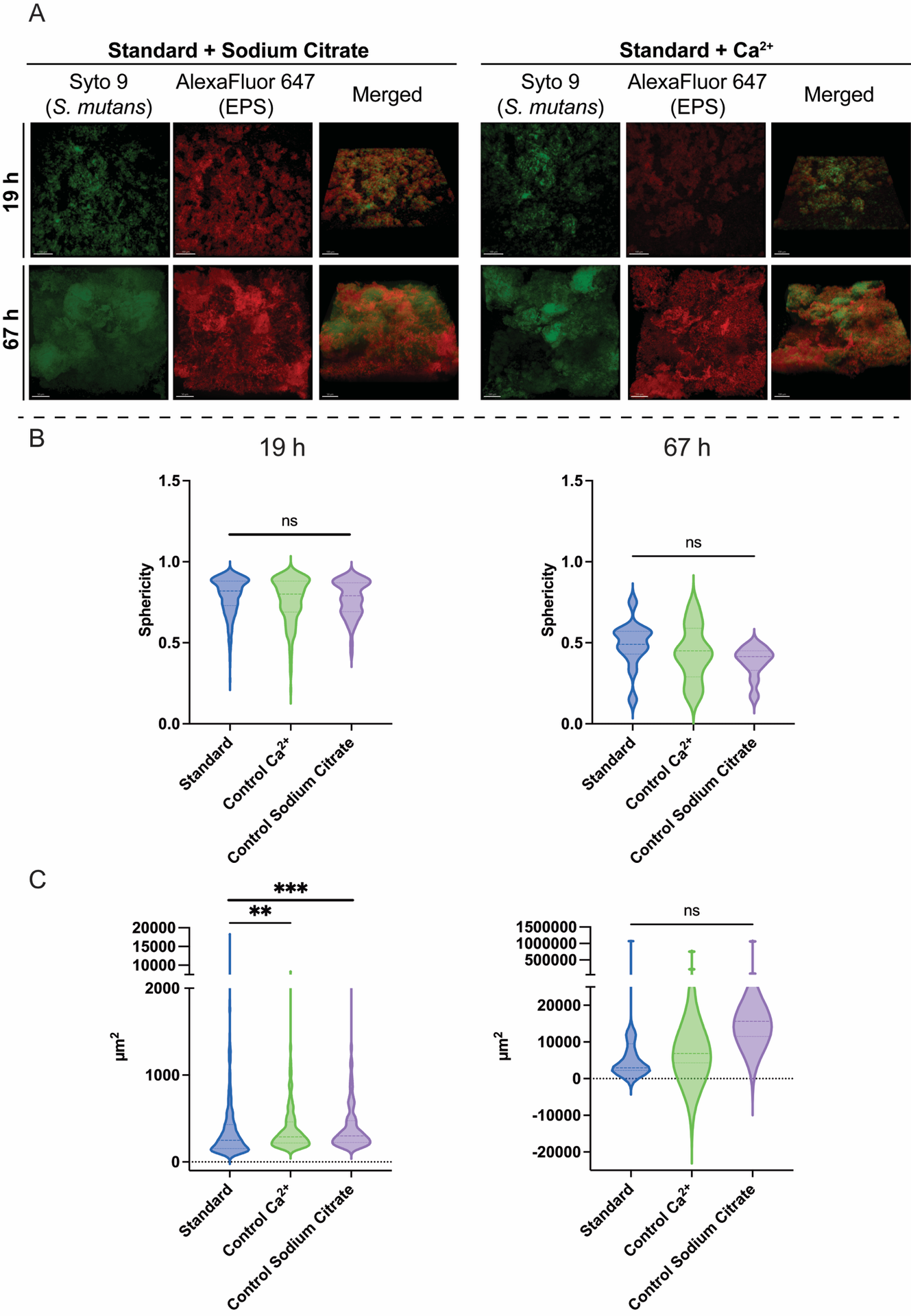


Supplementary Figure S4. Components of the bio-ink culture medium and bio-ink post-treatments do not affect spatial distributions of bacteria within standard *S. mutans* biofilms. Representative fluorescence confocal microscopy images of 19 h and 67 h *S. mutans* biofilms. The green color indicates microorganisms (labeled with SYTO9). The red color represents exopolysaccharides in the extracellular matrix produced by S. mutans (labeled with Dextran-Alexa Fluor 647). Single-channel images are top views. All merged images overlay both components and are tilted at 45°, with the HA surface located at Z=0 for each of the images. Imaging was performed at 20x. Scale bars represent 100 µm. B. Biofilm microcolony sphericity and C. Biofilm microcolony area (µm^2^) at 19 h and 67 h. Quantifications were performed using ImarisViewer 10.1.0. Statistical analyses were performed using a Kruskal-Wallis test followed by Dunn’s test. *** indicates p ≤ 0.001, ** indicates p ≤ 0.01, and ns indicates no statistical difference.


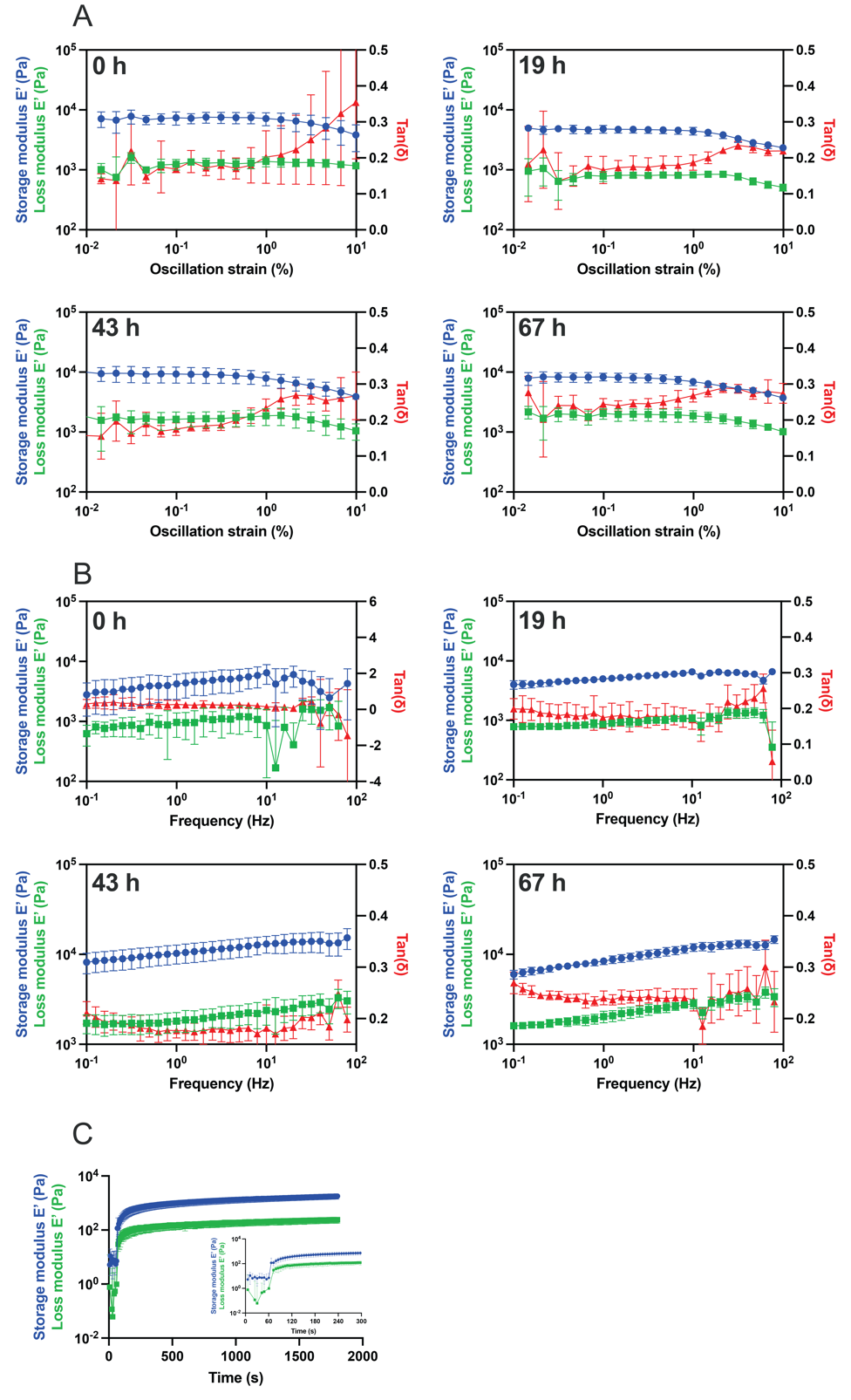


Supplementary Figure S5. Linear viscoelastic region (LVR) characterization of bio-ink biofilms. A. Strain sweep. B. Frequency sweep for bio-ink biofilms after 0, 19, 43, and 67 h of incubation, and C. Gelation for bio-ink biofilms from 0 to 30 min. At 1 min, 0.1M of calcium chloride was added. The inset graph displays the first 5 min of gelation. Blue circles represent storage modulus, green squares represent loss modulus, and red triangles represent tan-delta (n=5).
